# Evidence for causal links between education and maternal and child health: systematic review

**DOI:** 10.1111/tmi.13218

**Published:** 2019-03-28

**Authors:** Barbara S. Mensch, Erica K. Chuang, Andrea J. Melnikas, Stephanie R. Psaki

**Affiliations:** ^1^ Population Council New York NY USA

**Keywords:** infant and child health, education, low and middle‐income countries, maternal health, systematic review, santé du nourrisson et de l'enfant, éducation, pays à revenu faible et intermédiaire, santé maternelle, analyse systématique

## Abstract

**Objective:**

Numerous studies have documented an inverse association between years of schooling attained, particularly by women, and reduced maternal, infant and child mortality. However, if factors affecting educational attainment — many of which are unobservable, e.g. motivation and genetic endowment — also affect the likelihood of engaging in behaviours that enhance health, then assumed effects of schooling will be inflated in analyses that do not address this endogeneity. This systematic review assesses evidence for a causal link between education and maternal and child health in low and middle‐income countries.

**Methods:**

Eligible studies controlled for observable and unobservable factors affecting both education and health. Reported effects were converted into partial correlations. When possible, we also conducted meta‐analyses to estimate mean effects by outcome.

**Results:**

Of 4952 papers identified, 16 met the inclusion criteria. The 15 child health papers examined neonatal, infant and child mortality, stunting and wasting. Significant effects of education on infant and child health were observed for 30 of 33 models that did not account for endogeneity. In contrast, only 18 of 46 effects were significant in models that addressed endogeneity. Notably, for only one outcome —child mortality measured dichotomously —was the effect of maternal educational attainment significant in a meta‐analysis. The one maternal morbidity paper found significant effects for the two preventable outcomes considered.

**Conclusion:**

While we find evidence for a causal link between education and health, effects are weaker in models that address endogeneity compared to naïve models that do not account for unobservable factors affecting both education and health. Advances in women's educational outcomes have undoubtedly played a role in improving health in many settings; however, the effect is not as strong as some researchers and advocates have claimed.

## Abstract (fr)

**Objectif:**

De nombreuses études ont montré une association inverse entre le nombre d'années de scolarité atteintes, en particulier par les femmes, et la réduction de la mortalité maternelle, infantile et des nourrissons. Cependant, si les facteurs affectant le niveau d’éducation – dont beaucoup sont non observables, tels que la motivation et le patrimoine génétique – affectent également la probabilité d'adopter des comportements qui améliorent la santé, les effets supposés de la scolarité seront alors gonflés dans des analyses ne traitant pas de cette endogénicité. Cette analyse systématique évalue la preuve d'un lien de causalité entre l’éducation et la santé maternelle et infantile dans les pays à revenu faible ou intermédiaire.

**Méthodes:**

Les études éligibles contrôlaient les facteurs observables et non observables affectant à la fois l’éducation et la santé. Les effets rapportés ont été convertis en corrélations partielles. Dans la mesure du possible, nous avons également effectué des méta‐analyses pour estimer les effets moyens par résultat.

**Résultats:**

Sur 4.952 articles identifiés, 16 répondaient aux critères d'inclusion. Les 15 articles sur la santé infantile ont examiné la mortalité néonatale, infantile et du nourrisson, le retard de croissance et l’émaciation. Des effets significatifs de l’éducation sur la santé du nourrisson et de l'enfant ont été observés pour 30 des 33 modèles qui n'ont pas tenu compte de l'endogénicité. En revanche, seuls 18 des 46 effets étaient significatifs dans les modèles traitant de l'endogénicité. Notamment, pour un seul résultat – la mortalité infantile mesurée de manière dichotomique – dans une méta‐analyse, l'effet du niveau d’éducation de la mère était significatif. Le seul article sur la morbidité maternelle a révélé des effets significatifs sur les deux résultats évitables considérés.

**Conclusion:**

Bien que nous trouvons des preuves d'un lien de causalité entre l’éducation et la santé, les effets des modèles qui traitent de l'endogénicité sont plus faibles que ceux des modèles naïfs qui ne tiennent pas compte de facteurs non observables affectant à la fois l’éducation et la santé. Les progrès dans les résultats scolaires des femmes ont incontestablement joué un rôle dans l'amélioration de la santé dans de nombreux contextes; cependant, l'effet n'est pas aussi puissant que certains chercheurs et défenseurs le prétendent.

## Introduction

Numerous studies have documented an inverse association between years of schooling attained, particularly by women, and reduced maternal, infant and child mortality 1, 2, 3, 4, 5. It has been said that the link between education and child mortality is one of the strongest relationships established in public health 6. A systematic examination of trends in education and child health in 175 countries between 1970 and 2009 argued that about half the reduction in child mortality could be attributed to the increase in years of schooling of young women globally 6. Moreover, analyses of survey data from low income countries have shown that mother's education generally explains more of the variation in infant and child mortality than household economic resources 7, 8.

Mother's education is thought to be important for both preventing and treating poor health outcomes 9 and illness. A variety of pathways have been hypothesised linking education to maternal and child health including skill building, socialisation, information provision and delays in childbearing 10, 11, 12. Recently, literacy has become a focal point for theories about mechanisms linking female education to maternal and child health outcomes, serving both as a marker for skills gained in school and an indicator of future learning potential 13, 14. The contributions of literacy have been framed in terms of providing women with tools to learn more about the broader world as well as skills to acquire more targeted knowledge to improve child outcomes 15.

Maternal grade attainment and literacy are also associated with a wide range of preventive and treatment‐oriented health behaviours, including breastfeeding duration, well‐child care and effective use of health services 16. These health behaviours, in turn, have been found to partially mediate the relationship between women's schooling and child health as well as maternal health as use of health facility services for labour and delivery is considered by many to be the most effective strategy to reduce maternal deaths 2, 17, 18, 19, 20, 21, 22.

Researchers have argued that, in addition to developing academic skills and competencies that facilitate interaction with health bureaucracies, education potentially gives rise to more equitable gender attitudes and greater autonomy, which are likely gained through school‐based experiences and shifting expectations about the future 15. Although academic skills may facilitate their acquisition, changes in attitudes and autonomy may be more closely related to time spent in classrooms and social interactions between teachers and students, depending on the school environment 19. Better‐educated women are said to develop autonomy to act on health knowledge, navigate health institutions, control fertility and improve child health 14, 19, 23, 24, 25, 26, 27, 28, 29, 30, 31, 32, 33.

While a voluminous literature has established associations between education and health in developing countries, and while studies have referred to women's schooling as a ‘determinant’ of health 21, a question remains regarding the degree to which a causal relationship exists 34. Education is not randomly assigned. Those with more years of school are likely to be selective and, as such, factors affecting educational attainment such as motivation or genetic endowment may also affect the likelihood of engaging in behaviours that enhance maternal and child health 35. Yet claims have been made regarding the beneficial effects of education based on associations rather than through more rigorous methods to assess causality. Analyses that fail to address the potential endogeneity between health and schooling may lead to exaggerated conclusions about the degree to which education, in and of itself, is protective 2.

To document and fill this gap in knowledge, we conducted a systematic review of the evidence for a causal link between education and health in low and middle‐income countries. Establishment of causality is more than an academic exercise; it has implications for current policies and programs. If increases in educational attainment or improvements in skills acquired in school can be shown to reduce morbidity and mortality, then investments in education will likely have a direct payoff in terms of improved child and maternal health.

While the above discussion considers education broadly, the focus of this analysis is on mother's grade attainment and years of schooling because the vast majority of papers use these measures as the education exposure. However, we also summarise results from the few papers that consider father's education given interest in whether the effect is comparable to the effect for mother's education 36.

Note that this paper is one component of a two‐part effort to review the literature on the causal links between education and health. While the search included sexual and reproductive health and malaria, the results here are limited to those focused on maternal and child health. Findings from the review of the effects of education on sexual and reproductive health are summarised elsewhere (see Psaki, SR, *et al*. 2018) 37; no papers meeting the inclusion criteria were found for malaria. Details of the protocol for this systematic review were registered on PROSPERO and can be accessed here (https://www.crd.york.ac.uk/prospero/display_record.php?RecordID=73224;Registrynumber:CRD42017073224).

## Methods

### Search strategy

We searched select peer‐reviewed and gray literature databases (Box 1) for English language articles from 1990 or later using education as either an intervention or exposure. In addition to database searches, we reviewed reference lists for pertinent articles as well as recommended articles from the study advisory committee and authors. Search terms included measures of exposure to formal schooling as well as learning outcomes, and maternal and child health. Study design terms were also included. (Box 2). The search was conducted between July 30, 2017 and August 16, 2017. Authors and study advisors were given until December 18, 2018 to update data and provide any additional papers.

Box 1 **Databases searched**

PubMed, POPLINE, EconLit, ProQuest Dissertation Abstracts, Sociological Abstracts, WHO Regional (AIM), WHO Regional (LILACS), WHO Regional (WPRIM), WWW, DEC‐USAID, ELDIS, World Bank OKR, NBER Working paper series.

Box 2 **Examples of search terms**

- Measures of exposure to formal schooling: e.g. educational attainment, enrollment, school dropout, maternal education
- Measures of health: e.g. maternal morbidity and mortality, child stunting, child wasting, infant and child mortality, malaria.
- Study Design terms: e.g. randomisation, causality, experiment, instrumental variable and regression discontinuity.

### Inclusion/exclusion criteria

After the initial search, titles and abstracts were uploaded to Covidence, where two authors (2 and 3) reviewed titles and abstracts to assess relevance, including studies for full text review if their abstracts suggested that they met our inclusion criteria. Abstracts were assessed independently and disagreements were discussed before referring to a third author (4 or 1). Articles were included in full text review if they met the criteria listed in Box 3. Two authors (2 and 3) reviewed each full text. The complete list of inclusion and exclusion criteria is available in the registered protocol.

Box 3 **Inclusion criteria**

- Any of the exposure and outcome measures as noted in our study protocol.
- Same exposure(s) and outcome measure(s) were analysed for both treatment/quasi‐treatment and control/quasi‐control groups.
- Analysis attempted to control for the endogeneity between education and at least one health outcome of interest.
- Study methods eligible for inclusion were:
  o Randomised controlled trials (RCTs), both longitudinal and repeated cross‐sectional survey methods or natural experiments that addressed the endogeneity of schooling or other designs that addressed the endogeneity of policy changes or programs which improve schooling.
  o Quasi‐experimental studies with a controlled comparison.
- Reports on data from low‐ and middle‐income countries as defined by the World Bank.
- Published 1990 or later.
- Published in English.

Note: for an RCT or natural experiment to be included, the authors had to first show that it produced a positive effect on an education outcome. In the absence of a change in an education outcome, the authors would be unable to address the endogeneity between education and health.

### Data extraction

Data were single extracted. The data extraction form was designed in consultation with the Cochrane Handbook 38 and included study design; methods; participants; sample size; analysis methods; outcome measure(s); exposure measure(s); effect sizes; statistical significance; and discussion of mechanisms. In the course of data extraction, authors were contacted to request additional information as needed to compare study effect sizes.

### Grading of evidence

After data extraction, two authors (2 and 3) independently assessed study quality for each of the final included articles. Using an assessment tool adapted from Baird *et al*.^39^ and GRADE, studies were assigned points based on whether they addressed six domains: (i) selection bias; (ii) methods‐specific criteria (appropriate use and reporting of study design and analytical approaches); (iii) sample size; (iv) confounding factors; (v) respondent and data attrition; and (vi) mechanisms (authors describe any theoretical pathways linking education and health to motivate analyses). Each study was given 0 or 1 on the six criteria outlined, for a possible score ranging from 0 to 6.

### Quantitative analysis

In addition to presenting the coefficient estimates for both the ordinary least squares (OLS) models and the more rigorous models that addressed endogeneity, to summarise the results we designated the effect of education on a health outcome to be either ‘expected’ when the estimated effect of education was statistically significant and improved a health outcome, ‘null’ when the effect of education on a health outcome was not significant, and ‘unexpected’ when the effect of education was significant and led to a worse health outcome. Significance levels were based on *α* = 0.05, which may differ from results reported by some authors who use *α* = 0.10. For the synthesis, we use only measures of maternal education for our education exposures of interest because too few papers included paternal education.

To compare results across studies we converted reported effects into partial correlations (*r*) and provide 95% confidence intervals. We estimated the partial correlations for OLS models, when the authors provided estimates for such models, as well as for the more rigorous models. When there are at least three studies with the same exposure and outcome, we also compare effect sizes visually using forest plots with 95% CIs. The partial correlation has an absolute value of 1. When it is <0.2, which is the case for all papers considered here, the partial correlation is roughly equal to the standard deviation of the change in the health outcome associated with a one standard deviation change in the education variable controlling for, or ‘partialling out’, the other variables in the model 40 ^.^ Drawing on Cohen's 41 conventions for interpreting effect sizes, correlation effect sizes less than or equal to 0.10 are considered small, values of 0.25 are considered medium, and values greater than or equal to 0.40 are considered large 42. Typically, most systematic reviews that contrast groups compute standardised mean differences if the outcome is continuous and odds ratios if the outcome is dichotomous 42. However, because the final set of papers included multiple model types and because all papers incorporated complex estimation methods, the partial correlation was considered the most appropriate standardised effect size statistic. In some cases, e.g. linear models with a continuous or dichotomous education exposure, conversion was straightforward. But other conversions were more complex. See Data S1 for a more detailed description of the computation of partial correlations.

After converting each study's estimated effects to partial correlation coefficients, we conducted meta‐regressions to estimate mean effect sizes and 95% confidence intervals for outcomes when three or more studies were identified that (i) drew from different study populations; (ii) measured an exposure and outcome the same way; and (iii) used the same study design (i.e. quasi‐experimental or RCT). We ran random effects rather than fixed effects models based on the assumption that identified studies are drawn from a larger population of studies that do not have a common effect size^43^. We estimated a restricted maximum likelihood (REML) random effects model rather than the more‐often employed DerSimonian‐Laird (DL) model due to software limitations, but multiple studies have shown that DL and REML produce comparable estimates of between‐study variance under various conditions 44, 45, 46. Mean effect sizes were calculated by weighting each study's partial correlation coefficient by the within study variance using the following equation:

$$w_{i,\mathrm{RE}}=\frac{1}{\left(v_{i}+{\hat{\tau }}_{\mathrm{REML}}^{2}\right)}$$

where *w*_*i*_ is the weight of study *i*,* v*_*i*_ represents within‐study variance, and ${\hat{\tau }}_{\mathrm{REML}}^{2}$ is the between‐study variance for the REML model, as calculated below.

$${\hat{\tau }}_{\mathrm{REML}}^{2}=\max\left\{0,\frac{\sum w_{i,RE}^{2}\left({\left(y_{i}-{\hat{\mu }}_{\mathrm{RE}}\left({\hat{\tau }}_{\mathrm{ML}}^{2}\right)\right)}^{2}-v_{i}\right)}{\sum w_{i,\mathrm{RE}}^{2}}+\frac{1}{\sum w_{i,\mathrm{RE}}}\right\}$$

Here, *y*_*i*_ is the treatment effect for study *i*, ${\hat{\tau }}_{\mathrm{ML}}^{2}$ is the between‐study variance from the maximum likelihood model, and ${\hat{\mu }}_{\mathrm{RE}}$ is the mean effect size under a random‐effects estimation 47.

We report Cochran's *Q* statistic to assess the extent to which the included effect sizes all estimate the same population effect size (that is, from a homogenous population). Since the variation in effect sizes between studies may reflect both true heterogeneity and random error, a statistically significant *Q* indicates that the variation in study effect sizes is not due to error alone, and therefore studies are drawn from a heterogeneous population. However, a non‐significant *P*‐value for Q does not necessarily rule out the possibility of variations in true effects, as this may be due to low power, which is likely when there are few studies 43. We also report the between‐study variance estimator *τ* 2, as calculated above from the REML model. In addition, we include the *I* ^2^ statistic, a study‐level measure of effect, which quantifies the percentage of the variance in a set of studies that is due to the studies themselves rather than sampling error. Cochrane collaboration guidelines suggest that an *I* ^2^ greater than 75% indicates considerable heterogeneity between studies, while a value below 40% indicates that heterogeneity may be unimportant 48. Given that there are few studies included in each regression we do not conduct a moderator analysis which is used to investigate factors causing the heterogeneity.

### Narrative synthesis

We begin the summary of results by first providing a description of the papers, including the type of publication, the country, the sample size, the type of analyses the authors conducted, the estimation procedures and our assessment of the risk of bias. We organise the quantitative analysis by outcome beginning with neonatal, infant and child mortality, then turning to child stunting and wasting, and then finally considering maternal morbidity. We summarise the papers in tabular form for each health outcome separately converting each study's result into a common statistic. While we calculate the frequency of different results across studies, specifically the extent to which significant results in ‘naïve’ analyses are retained in more rigorous models, our quantitative synthesis is not limited to ‘vote counting’ but also, where possible, includes meta‐analysis. We then consider the effect of father's educational attainment for the few papers that analyse this exposure. Finally, to the extent that authors discuss mechanisms, we have summarised their findings about the pathways linking education to health.

## Results

### Study selection

Of the 4952 papers identified, 16 were included in this review. Half were published in peer reviewed journals, and half were working papers. All studies focused on a single country; half analysed data from sub‐Saharan Africa, and the other half analysed data from Asia, the Middle East/North Africa and Latin America. Eleven of the 16 papers used data from the Demographic and Health Surveys (Table 1).

**Table 1:** Study characteristics

Authors (year)	Publication type	Country	Sample size	Type of analysis	Estimation procedure	Risk of bias score
Ali & Elsayed (2018) 51	Journal article	Egypt	95,191 mothers; 345,235 children	Quasi‐experimental	2SLS, RDD	3
Baird, McIntosh, & Ozler (2018) 56	Working paper	Malawi	2049 females	RCT	OLS	6
Breierova & Duflo (2003) 36	Working paper	Indonesia	122,818 mothers; 98,953 sons; 96,391 daughters	Quasi‐experimental	2SLS, OLS	3
De Neve & Subramanian (2017) 57	Journal article	Zimbabwe	8243 children	Quasi‐experimental	2SLS, OLS	4
Dinçer, Kaushal, & Grossman (2014) 35	Journal article	Turkey	5233 females	Quasi‐experimental	2SLS, OLS	5
Dursun, Cesur, & Kelly (2017) 54	Working paper	Turkey	1,486,353 females; 340,091 children	Quasi‐experimental	2SLS, OLS	5
Fazlul (2018) 58	Working paper	Bangladesh	19892 women	Quasi‐experimental	2SLS, OLS	2
Grépin & Bharadwaj (2015) 49	Journal article	Zimbabwe	7813 females	Quasi‐experimental	2SLS, OLS	3
Güneş (2015) 59	Journal article	Turkey	1677 ever‐married females	Quasi‐experimental	2SLS, OLS	4
Keats (2018) 55	Working paper	Uganda	12966 females	Quasi‐experimental	2SLS	3
Makate & Makate (2016) 50	Journal article	Malawi	20,299 mothers; 67,225 toddlers	Quasi‐experimental	2SLS, OLS	4
Makate (2016) 52	Journal article	Uganda	9957 females; 34,102 children	Quasi‐experimental	2SLS, OLS	4
Maïga (2011) 60	Working paper	Burkina Faso	2007 children	Quasi‐experimental	2SLS, OLS	3
Shrestha (2016) 53	Working paper	Nepal	3349 mothers (literacy), 3306 mothers (grade attainment)	Quasi‐experimental	2SLS, OLS	4
Tequame & Tirivayi (2015) 61	Working paper	Ethiopia	4291 females	Quasi‐experimental	2SLS, OLS	4
Weitzman (2017) 64	Journal article	Peru	5441 females	Quasi‐experimental	2SLS, OLS	3

**Figure 1:**
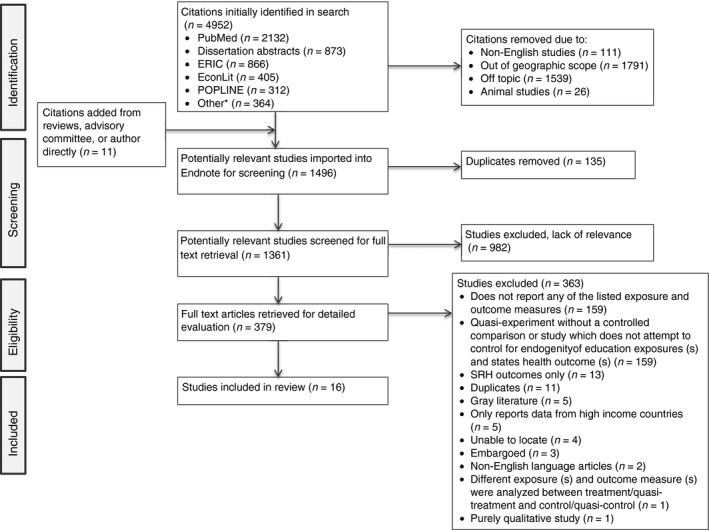
PRISMA flow diagram MCH.

Figure 1 shows the PRISMA flow diagram. Of the final set of 16 papers, only one study investigating maternal morbidity, and no papers on maternal mortality or malaria, met our inclusion criteria. Of the papers that examined child health, seven investigated the effects of education on both infant and child mortality 35, 36, 49, 50, 51, 52, 53, one measured child but not infant mortality separately 54 and one measured infant but not child mortality 55. Of the nine papers that investigated infant mortality [35, 36, 49, 50, 51, 52, 53, 55, 56], five also examined neonatal mortality 35, 36, 51, 52, 56. Of the papers that measured child growth faltering, eight investigated child stunting 51, 55, 56, 57, 58, 59, 60, 61 and, of these, five also measured child wasting 51, 55, 57, 58, 60 and three 51, 55, 58, 59 measured child weight for age 55, 58, 59.

### Health outcomes

The outcome measures differed across studies. Many infant and child health studies had continuous outcomes: for growth faltering these included *z*‐scores and number of children stunted before age 5 based on World Health Organization definitions; for infant and child mortality these included number of children dead before one month, one year or five years and mortality rates. Other child health studies had dichotomous measures: any stunted or wasted child and infant or child death by the survey. While not all authors were specific about whether the child mortality measure was inclusive of infant mortality, that is whether the child mortality results refer to 4q1 rather than 5q0, or whether infant mortality was inclusive of neonatal mortality, based on the wording provided, all papers that assessed infant or child mortality included younger age brackets except for Baird, McIntosh and Özler's (2018) 56 measure of infant mortality, which excluded neonatal mortality. For the paper investigating maternal morbidity, all measures were dichotomous: ever experienced fever, excessive vaginal bleeding or convulsions during pregnancy and mastitis after childbirth. While we will discuss the results for wasting and maternal morbidity outcomes since they were included in our search terms, tables summarising results from these models are provided in a supplementary section ([Link], [Link], [Link], [Link]) rather than the main paper because wasting is considerably less common than stunting 62, 63 and is often short term since it is generally the consequence of an acute food shortage 62, 63, and because only one paper was available for maternal morbidity.

### Education exposure

Of the 16 papers, 11 measured the effects of maternal grade attainment only (i.e. years of school completed either as a continuous or dichotomous measure); one measured grade attainment and literacy, three measured years of schooling, which could differ from years attained because of grade repetition and one, focused on adolescents, measured school attendance. With a few exceptions, studies did not measure whether the effect of maternal schooling is linear or whether there are threshold effects, that is whether, for example, the positive effect of schooling on health is realised with primary or secondary completion. Because the measure of education is continuous for most studies, where forest plots are provided, treatment/exposure and control group numbers are not provided. While all 16 papers considered the effect of maternal education, three papers 36, 51, 57 also considered the effect of paternal educational attainment on child health.

### Study design

Only one paper 56 analysed data from a randomised controlled trial. Fifteen of the 16 studies took advantage of natural experiments, e.g. school construction program in Indonesia 36, implementation of universal primary education policies (elimination of primary school fees) in Malawi 50 and Uganda 52, 55, increases in years of compulsory education in Peru 64 and Turkey 35, 54, 59, and a secondary school scholarship program in Bangladesh 58. These 15 papers all used exposure to the intervention or policy change as an instrument to address the endogeneity in the relationship of interest. That is, they exploited the variation in the intensity of, or exposure to, the reform across cohorts, which resulted in exogenous variation in the supply of schooling to mimic a randomised controlled experiment. Instrumental variables (IV) estimation is widely used in economic research to assess causality with observational data when correlation between the explanatory variable and the error term is suspected due, for example, to an omitted variable 65. The goal is to identify an observable variable that induces changes in the endogenous explanatory variable, in this case education, but has no direct effect on the dependent variable, in this case, health. That is, the instrument is supposed to influence health only through its effect on education. Two stage least squares (2sls) models are then estimated. In the first stage, the endogenous explanatory variable, education, is regressed on the exogenous explanatory variables in the model as well as the excluded instrument. To obtain unbiased estimates of the effect of education, in the second stage the endogenous school attendance variable is replaced with the predicted values from the first‐stage estimates. For this estimation technique to perform well, that is for hypothesis testing based on IV estimates to be correct, the instrument most be valid. There must be a convincing theoretical argument ruling out any direct effect of the instrument on the dependent variable at the same time the instrument must be correlated with the endogenous regressor. If these conditions are not satisfied the instrument is considered to be weak and 2sls may introduce more bias than the ordinary least squares model it is intended to replace. The advantage of a natural experiment is that these conditions can often be met, that is ‘there is usually a well‐developed story or model motivating the choice of instruments’ 66.

### Risk of bias

In our ‘risk of bias’ assessment, while only one study scored a five or six 56, given the stringent criteria for this review, we consider all papers that met our inclusion criteria to be higher quality than much of the existing literature. The scores assigned are a function of the level of detail reported by the authors, rather than fundamental flaws in study design. (Table S2). Both because only 16 studies met our stringent criteria and limited variability in analytic quality, we do not separate analyses by scores, although in the tables we do indicate studies that received a score of 4 or higher.

### Infant and child health

Of the 14 quasi‐experimental papers that investigated infant and child health, 12 compared OLS models with more rigorous models that addressed endogeneity. These 12 papers examined 33 outcomes, 17 of which are unique. (A list of the 17 outcomes is included as Table S1). Significant effects of education on health were found for 30 of 33 outcomes in OLS models, seven of nine for neonatal and infant mortality 35, 36, 49, 50, 52, 53, 12 of 12 for child mortality 35, 36, 49, 50, 52, 53, 54 and 11 of 12 for growth faltering 57, 58, 59, 60, 61. Only 16 of the 33 outcomes were significant in the more rigorous models for papers comparing OLS to these more rigorous models; five of nine for infant and neonatal mortality 36, 49, 50, 52, 53, eight of 12 for child mortality 36, 49, 50, 52, 54 and two of 12 for growth faltering 55, 58. In summary, while there is evidence for a causal link between education and child health, effects are considerably weaker in models that address endogeneity by comparison to naïve models. The discussion below considers all papers separately by outcome, not just those that compared OLS models with more rigorous models. In total, the 15 infant and child health papers examined 46 outcomes, 18 of which were significant in models that addressed endogeneity. The tables — both those included in the main paper as well as the supplement— separate results by the approach used by authors to measure the outcome.

### Neonatal, infant and child mortality

The effect of maternal education was significant in the expected direction for five 36, 49, 50, 52, 53 of 12 infant mortality models (Table 2.3–2.5) and eight 36, 49, 50, 52 of 13 child mortality models (Table 33.1–3.6). Only one paper 53 that included both infant and child mortality as an outcome found a significant effect for one outcome, infant mortality, and a null effect for child mortality. Consistent with early descriptive research 67, the evidence for an effect of education on neonatal mortality (Table 2.1 and 2.2) is weaker than for infant and child mortality; only one 36 of the four papers found a significant effect with more rigorous models. Although the signs calculated for the partial correlations are always consistent with the signs for the coefficients produced by the authors, there is one infant and child mortality paper 52 where the significance level reported (equivalent to *α* = 0.05) for infant mortality does not correspond with the 95% CI that we calculated. There is insufficient information reported by the author to account for the discrepancy.

**Table 2:**
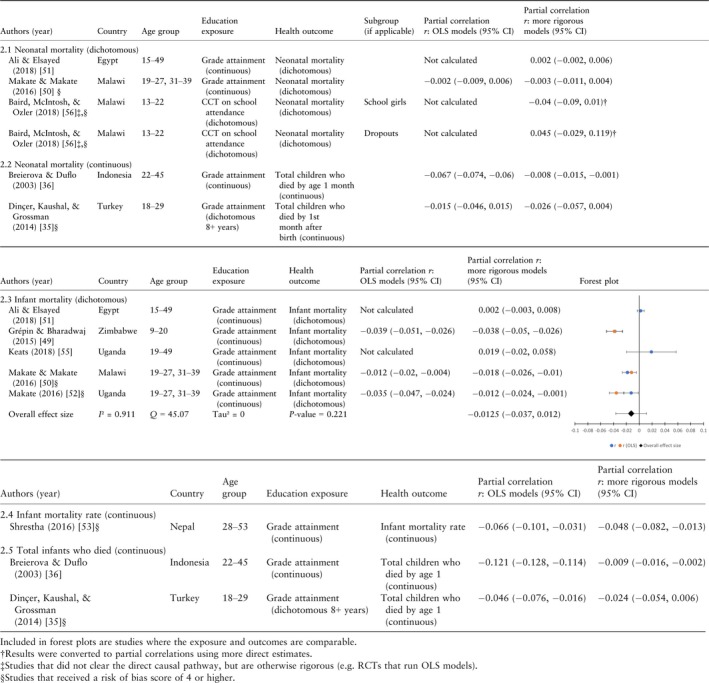
Results for neonatal and infant mortality

**Table 3:**
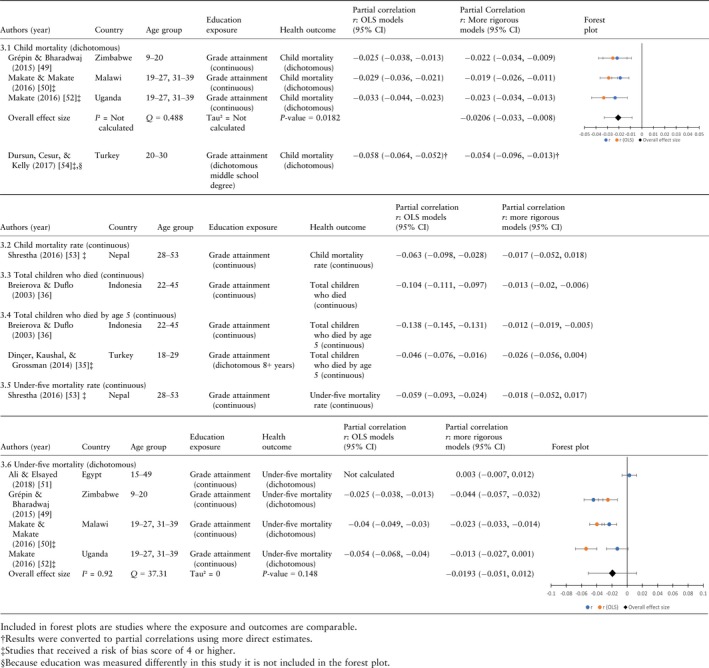
Results for child mortality

### Stunting and wasting

The results for the child growth faltering models —stunting, wasting, underweight— varied, but apart from stunting the number of papers identified for each of these outcomes is very small. For stunting, which reflects cumulative nutritional deprivation and infection, three measures were used: (i) height‐for‐age *z*‐score (HAZ) where we expect a positive association with education (Table 4.1), (ii) a dichotomous indicator of stunting where height‐for‐age is more than two standard deviations below the WHO Child Growth Standards median (Table 4.3), and (iii) total number of stunted children under age five, again based on the WHO standard; for the latter two we expect a negative association (Table 4.2). For wasting, which reflects acute undernutrition, there were two measures: (i) weight‐for‐height *z*‐score (WHZ), where we expect a positive association with education, and (ii) a dichotomous indicator of wasting where weight‐for‐height is more than two standard deviations below the WHO Child Growth Standards median, for which we expect a negative association with education. For underweight, which reflects both chronic and acute malnutrition, there is one measure, weight‐for‐age *z*‐score (WAZ), for which we expect a positive association with education ([Link], [Link], [Link], [Link]).

**Table 4:**
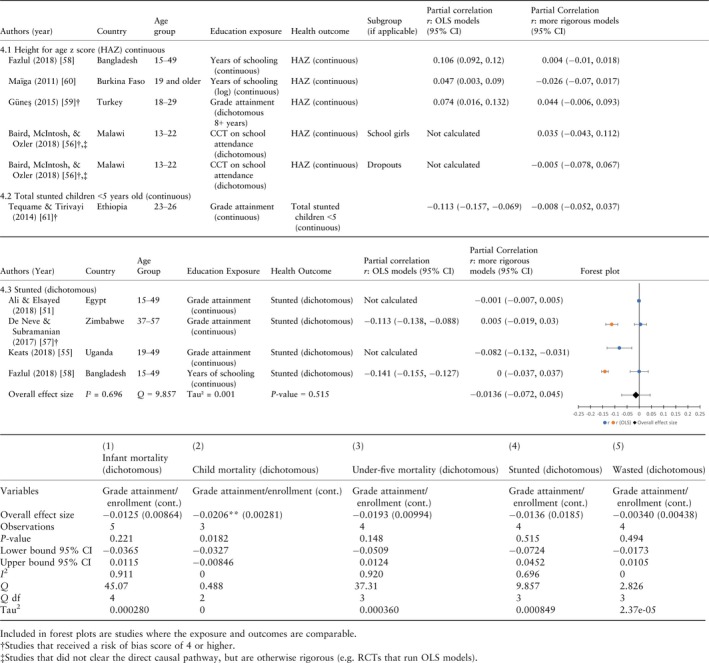
Stunting

The effect of maternal education was significant in the expected direction in more rigorous models for two of ten child stunting outcomes^55^, two^58^, ^60^ of six child wasting outcomes and none of the three weight‐for‐age outcomes. While there are too few papers examining the effect of education on child growth faltering to draw definitive conclusions, the few that satisfied our inclusion criteria did not have consistent findings across outcomes. One paper that found a significant effect on stunting in Uganda 55 did not find a significant effect on wasting whereas a paper analysing data from Burkina Faso 60 found the opposite pattern (see Table 4.1–4.5). For one paper analysing the effect of education on stunting 59, the value of r was not significant, but the coefficient calculated by the author was. (Note, in another paper Fazlul (2018) indicates significant effects of education in 2sls models for three outcomes: wasting, WAZ and WHZ, but only WHZ was significant in our analysis because we set it alpha = 0.05 whereas he set alpha = 0.1.)

### Meta‐analysis

Given the small number of studies and the criteria listed above, we were only able to conduct meta‐regressions for five of the 17 infant and child health outcomes in seven papers 49, 50, 51, 52, 55, 57, 58. Consistent with that observed in the forest plots, meta‐regression results indicate a statistically significant mean effect size of increased grade attainment only on child mortality measured dichotomously: −0.0206 (*P* = 0.018). While three of the five meta regressions indicate that *I* ^2^ is high — indicating substantial heterogeneity between studies — for child mortality measured dichotomously it is zero. However, the *I* ^2^ index has low statistical power when the number of studies is small, and thus no definitive conclusions should be drawn about heterogeneity for the child mortality model 68. Suffice it to say: based on the results from three of the other regressions as well as the information available about the characteristics of each study, it is likely that there is considerable between‐study variability related to the samples and the study designs thus limiting our ability to make definitive statements about the magnitude of the significant relationship between maternal education and child mortality and the source of heterogeneity (Table 5).

**Table 5:** Meta‐analysis

	(1)	(2)	(3)	(4)	(5)
	Infant mortality (dichotomous)	Child mortality (dichotomous)	Under‐five mortality (dichotomous)	Stunted (dichotomous)	Wasted (dichotomous)
Variables	Grade attainment/enrollment (cont.)	Grade attainment/enrollment (cont.)	Grade attainment/enrollment (cont.)	Grade attainment/enrollment (cont.)	Grade attainment/enrollment (cont.)
Overall effect size	−0.0125 (0.00864)	−0.0206** (0.00281)	−0.0193 (0.00994)	−0.0136 (0.0185)	−0.00340 (0.00438)
Observations	5	3	4	4	4
*P*‐value	0.221	0.0182	0.148	0.515	0.494
Lower bound 95% CI	−0.0365	−0.0327	−0.0509	−0.0724	−0.0173
Upper bound 95% CI	0.0115	−0.00846	0.0124	0.0452	0.0105
*I* ^2^	0.911	0	0.920	0.696	0
*Q*	45.07	0.488	37.31	9.857	2.826
*Q* df	4	2	3	3	3
Tau^2^	0.000280	0	0.000360	0.000849	2.37e‐05

Standard errors in parentheses.

** *P* <0.05.

### Maternal morbidity

The one study that investigated maternal morbidity found significant effects in the expected direction for outcomes that were designated as preventable in the more rigorous models 64 (Table S6). However, as was the case for child health, while signs are always consistent for *r* and the author's coefficients, there are discrepancies between the author's calculations of significance and the significance levels calculated for partial correlations; this occurred for excessive bleeding during pregnancy and convulsions.

### Mother's versus father's educational attainment

It has been hypothesised that mother's educational attainment matters more for child health outcomes than father's because in most developing country settings it is the women who are primarily responsible for childcare. As noted above, only three of the 15 child health papers 36, 51, 57 included results examining the effect of mother's and father's educational attainment, and only one 36 compared the magnitude of the effects. The results for the two papers that investigated the effect of educational attainment on both infant and child mortality were similar for mothers and fathers; that is, one paper found significant and equivalent size effects of grade attainment for mothers and fathers 36 and the other observed insignificant effects for both mothers and fathers 51. Another paper 50 mentioned estimating models that examined the effect of paternal education on child mortality but did not show results. The paper 50 reported significant effects for mothers but not for fathers. Two of the papers investigated child stunting 51, 57. One found a null effect for mothers but a weak significant effect for fathers (at *α* = 0.10) 51, and the other found a null effect for both mothers and fathers 57. Finally, one paper considered wasting and found a null effect for both mothers and fathers 51.

### Hypothesised mechanisms linking education and maternal and child health

Given the strength of the association between maternal education and child health, demographers have speculated about ‘the pathways of influence 2.’ Most of the papers included in this review explored mechanisms, either theoretically or empirically (Tables S7–S9). However, the analyses typically focused on whether education influenced the particular mechanism, not whether that mechanism significantly affected health. Pathways explored included the effects of education on: sexual and reproductive behaviour, health behaviours, maternal health status, resources and knowledge, skills and attitudes. It is beyond the scope of this review to investigate which mechanisms find the most empirical support as that would require a systematic analysis of all papers exploring those relationships, a much larger sample of papers than discussed in this review. However, for the nine child health papers that investigate mechanisms empirically, few consistent pathways emerge, which in part may reflect that data were not available to analyse the same set of mechanisms across studies. The one consistent intervening pathway linking maternal education to child health outcomes is reproductive behaviour. However, our review of the sexual and reproductive health literature did not find consistent evidence of mechanisms linking education and those outcomes (see Authors 2018 37) leaving us with an incomplete picture of whether it is knowledge, skills, or attitudes and preferences that underlie the associations.

## Discussion

To the best of our knowledge, this is the first systematic review of rigorous studies attempting to address the endogeneity between education and maternal and child health in low and middle‐income countries. While significant effects of education on infant and child health were observed for 30 of 33 OLS models that did not address endogeneity, only 16 of the 33 effects were significant in more rigorous models. Including all 15 child health papers, not just those comparing OLS to more rigorous models, we examined 46 models, only 18 of which were significant when endogeneity was addressed. This analysis has demonstrated that models that do not address the shared factors driving the association between maternal education and infant and child health considerably overstate the effect of education. While papers published in journals or as working papers are often biased in favor of reporting significant effects of education on maternal and child health 69, one characteristic of the papers included in this review argues against that: almost all authors had the goal of demonstrating whether significant effects found in OLS models were maintained in models addressing endogeneity; thus if insignificant effects were found in the more rigorous models, that was a worthy finding in and of itself.

Given the small number of papers per outcome we were only able to conduct meta‐analysis for five of 17 outcomes. Notably for only one outcome — child mortality measured dichotomously — is maternal educational attainment significant. Why might mother's education have a more consistent effect on child mortality than on neonatal and infant mortality and stunting? There are several potential explanations: (i) For almost all papers the measure of child mortality included infant deaths, and thus there was more power to detect an effect of education for child mortality than for neonatal and infant mortality models. (ii) Many neonatal and infant deaths are caused by congenital malformations and genetic abnormalities (in addition to obstetric care and delivery complications), whereas deaths after age one are more often a result of environmental conditions, sanitation, nutrition, immunisation and child care practices 70. These so‐called exogenous or external factors are more likely to be influenced by maternal education 9. (iii) In addition to recurrent infections, stunting is caused by inadequate nutrition 71, which is likely more a function of financial resources of the household than of education of the mother.

This review had several limitations. First, although we calculated mean effect sizes for each individual paper and overall mean effects for selected outcomes via meta‐regressions, the small number of studies per outcome and the considerable variability in number and type of variables included in each model, even in studies with similar designs, makes it difficult to draw definitive conclusions about the magnitude of the effect of education on health. Because the 15 child health papers investigated 17 different outcomes, we summarised the results by counting significant effects and, where possible, compared the OLS results to the results where endogeneity was addressed. Yet there are serious limitations to ‘vote counting’ in that every study is treated equivalently, which means that the magnitude of the effect, the sample and the study quality are not considered. The Cochrane review guidelines note that ‘Vote counting might be considered as a last resort in situations when standard meta‐analytical methods cannot be applied (such as when there is no consistent outcome measure 38. What we have noted above and can conclude with confidence based on both the counting procedure used in all 15 child health papers and the meta‐analyses for seven of the papers is that the effect of maternal education on health, which appears to be significant in almost all OLS models, is attenuated in models that account for endogeneity. We recommend that future studies investigating the effect of education on child health include outcome measures that are most commonly used, which will facilitate comparisons between studies and make it possible to conduct meta analyses with more studies. Specifically, we suggest that researchers measure child health with dichotomous outcome variables, i.e. ever neonatal, infant and child death and stunted and wasted (yes/no).

Second, although there is considerable heterogeneity between studies included in this review, we are unable to conduct moderator analyses that would help explain the source of heterogeneity. For example, it might be expected that the magnitude of the effect of maternal schooling on health would depend, in part, on the overall level of education in the country or geographic area under consideration. For example, education might be a more important determinant of child health in settings of extreme poverty, substituting for non‐existent or poor‐quality health facilities, and inadequate water and sanitation infrastructure. Alternatively, mother's education might matter more where health facilities are accessible and of reasonable quality as the better educated may be more able to take advantage of such facilities. The 15 child health papers are based on data from nine countries for which the mean years of schooling varies from 1.5 (Burkina Faso) to 8.1 (Zimbabwe) 72.While there is no evidence from our analysis that the presence of significant effects varies consistently with the overall level of school, the number of countries is insufficient to draw any definitive conclusions.

Third, almost all papers focused on grade attainment or years of schooling as the education exposure, likely due not only to data limitations, but also because they exploited natural experiments, primarily in the form of policy or programmatic changes designed to increase access and/or attainment. This undermines our ability to identify what aspect(s) of schooling improves maternal and child health. Is it the challenging of traditional notions of disease and acquisition of specific preventative and curative procedures through life skills curricula? Is it improvement of literacy that provides the tools to acquire and use targeted information 13? Is it that the process of learning produces structural changes in the brain thereby enhancing cognitive abilities associated with improved health behaviours 7? Is it the development of autonomy that allows individuals, especially women, to act on health knowledge and navigate health institutions 15? The absence of an array of measures means that grade attainment will likely capture the effects of other dimensions of schooling that are potentially key for improving health outcomes.

Fourth, very few papers investigated the effect of both mothers’ and fathers’ grade attainment, and thus it is not possible to draw conclusions on whether there is a gender difference in the effect of parental education on child health. Fifth, no paper examined the relationship between education and maternal mortality, a relatively rare event compared to other outcomes studied, and only one examined maternal morbidity, for which data are not often collected. Sixth, the exclusion of papers prior to 1990 and the restriction to studies published in English may have led us to overlook a few papers, although it is our understanding that the vast majority of papers on this subject are published in English and efforts to address the endogeneity are recent.

While this review provides evidence of significant effects of maternal grade attainment on key health outcomes in the expected direction in a number of settings and while our meta analyses point to a significant effect of maternal education on child mortality, it is worth investigating why null effects were observed in a number of models. Three papers discussed null effects and provide insights that might be relevant in settings beyond those particular papers. The following reasons were mentioned: (i) secondary schooling was not sufficient to reduce child stunting (Zimbabwe) 57; (ii) vaccination coverage was extensive (Ethiopia), reducing the possibility of finding an effect of education on child health; and (iii) school quality (Egypt) was too poor to expect a strong effect on child health. More research on the pathways might shed light on when and why women's education is not always a significant factor in improving health.

Rapid expansion of school participation in many low‐income countries since the 1990s was motivated in part by the promise of improving health outcomes. Given that many initiatives to expand schooling access have not resulted in substantially improved learning, it is important to examine whether positive effects of schooling on health outcomes persist in low school quality contexts in order to guide effective education investments. Increases in the educational attainment of women have undoubtedly played a role in improving child health, and likely maternal health, in low and middle‐income countries. However, as this review demonstrates, the effect is not nearly as strong as some researchers and advocates have claimed. The effects of increases in maternal grade attainment on infant and child health are smaller than those observed in naïve analyses that fail to address endogeneity. This is not unexpected given that improvements in both education and health share a common set of determinants, including poverty and quantity and quality of infrastructure. Moreover, to the extent that many improvements in health, e.g. immunisation campaigns, are experienced by even the poorest and least educated members of society, we might not observe large variability in certain health outcomes by educational attainment. In addition, the effect of education on health may diminish over time due to declining school quality, a consequence of the rapid increase in school enrollment in many settings. Indeed, variability across studies in the effect of grade attainment on health observed currently may be due to differences in school quality. If, however, it is merely participation in school that boosts young women's ability and inclination to engage with the health sector, academic skill acquisition may not be as important as school participation. If so, perhaps whatever beneficial effect of education that currently exists will persist regardless of school quality 6. Future research should examine the independent effects of school enrollment, grade attainment and learning on health and investigate the mechanisms underlying each of these effects.

## Supporting information


**Table S1.** List of outcomes in the 15 infant and child health papers.

**Table S2.** Risk of bias assessment results by study.

**Table S3.** Wasted (dichotomous).

**Table S4.** Weight for Height Z score continuous (WHZ).

**Table S5.** Weight for Age Z score continuous (WAZ).

**Table S6.** Maternal Morbidity.

**Table S7.** Evidence in support of hypothesised mechanisms linking grade attainment and infant mortality.
**Table S8.** Evidence in support of hypothesised mechanisms linking grade attainment and child mortality.
**Table S9.** Evidence in support of hypothesised mechanisms linking grade attainment and child growth faltering.

**Data S1.** Model types and formulas used for conversion to partial correlations.

## References

[1] Pamuk ER , Fuchs R , Lutz W . Comparing relative effects of education and economic resources on infant mortality in developing countries. Popul Dev Rev 2011: 37: 637–664. 22319768 10.1111/j.1728-4457.2011.00451.x

[2] Cleland JG , van Ginneken JK . Maternal education and child survival in developing countries: The search for pathways of influence. Soc Sci Med 1988: 27: 1357–1368. 3070762 10.1016/0277-9536(88)90201-8

[3] Bhalotra S , Clarke D . Educational attainment and maternal mortality: UNESCO, 2013.

[4] Hobcraft JN . Women's education, child welfare and child survival: a review of the evidence. Health Transit Rev 1993: 3: 159–175. 10146571

[5] Mensch BS , Lentzner H , Preston S . Socioeconomic Differentials in Child Mortality in Developing Countries. 1985; ST/ESA/SER.A/97.

[6] Gakidou E , Cowling K , Lozano R , Murray CJL . Increased educational attainment and its effect on child mortality in 175 countries between 1970 and 2009: A systematic analysis. Lancet 2010: 376: 959–974. 20851260 10.1016/S0140-6736(10)61257-3

[7] Lutz W , Kebede E . Education and Health: Redrawing the Preston Curve. Popul Dev Rev 2018: 44: 343–361. 29937609 10.1111/padr.12141 PMC6001628

[8] Fuchs R , Pamuk E , Lutz W . Education or wealth: which matters more for reducing child mortality in developing countries? Vienna Yearb Popul Res 2010: 8: 175–199.

[9] Infant PA , Mortality C . In: Demeny P McNicoll G (eds). The Encyclopedia of Population. New York, USA: Macmillan Reference, 2003, 533–536.

[10] Rowe ML , Thapa BK , Levine R , Levine S , Tuladhar SK . How does schooling influence maternal health practices? Evidence from Nepal Comp Educ Rev 2005: 49: 512–533.

[11] Basu A , Stephenson R . Low levels of maternal education and the proximate determinants of childhood mortality: A little learning is not a dangerous thing. Soc Sci Med 2005: 60: 2011–2023. 15743650 10.1016/j.socscimed.2004.08.057

[12] Glewwe P . Why does mother's schooling raise child health in developing countries? Evidence from Morocco J Hum Resour 1999: 34: 124–159.

[13] LeVine RA , LeVine S , Schnell‐Anzola B , Rowe ML , Dexter E . Literacy and Mothering: How Women's Schooling Changes the Lives of the World's Children. Oxford University Press: New York, 2012.

[14] Smith‐Greenaway E . Mothers’ reading skills and child survival in Nigeria: examining the relevance of mothers’ decision‐making power. Soc Sci Med 2013: 97: 152–160. 24161100 10.1016/j.socscimed.2013.08.011 PMC3816368

[15] Jejeebhoy S . Women's Education, Autonomy, and Reproductive Behaviour: Experience from Developing Countries. Clarendon Press: Oxford, 1995.

[16] Smith LC , Ramakrishnan U , Ndiaye A , Haddad L , Martorell R . The Importance of Women's Status for Child Nutrition in Developing Countries. International Food Policy Research Institute and Emory University: Washington, DC, 2003.

[17] Semba RD , de Pee S , Sun K , Sari M , Akhter N , Bloem MW . Effect of parental formal education on risk of child stunting in Indonesia and Bangladesh: a cross‐sectional study. Lancet 2008: 371: 322–328. 18294999 10.1016/S0140-6736(08)60169-5

[18] Frost MB , Forste R , Haas DW . Maternal education and child nutritional status in Bolivia: Finding the links. Soc Sci Med 2005: 60: 395–407. 15522494 10.1016/j.socscimed.2004.05.010

[19] Joshi AR . Maternal schooling and child health: preliminary analysis of the intervening mechanisms in rural Nepal. Health Transit Rev 1994: 4: 1–28. 10147162

[20] Das GM . Death clustering, mothers’ education and the determinants of child mortality in rural Punjab, India. Popul Stud 1990: 44: 489–505.

[21] Filippi V , Ronsmans C , Campbell OM *et al* Maternal health in poor countries: the broader context and a call for action. Lancet 2006: 368: 1535–1541. 17071287 10.1016/S0140-6736(06)69384-7

[22] Campbell OM , Graham WJ , Group LMSSs . Strategies for reducing maternal mortality: getting on with what works. Lancet 2006; 368: 1284–1299. 17027735 10.1016/S0140-6736(06)69381-1

[23] Vikram K , Vanneman R , Desai S . Linkages between maternal education and childhood immunization in India. Soc Sci Med 2012: 75: 331–339. 22531572 10.1016/j.socscimed.2012.02.043 PMC3495071

[24] Cassell JA , Leach M , Fairhead JR , Small M , Mercer CH . The social shaping of childhood vaccination practice in rural and urban Gambia. Health Policy Plan 2006: 21: 373–391. 16940303 10.1093/heapol/czl020

[25] Babalola A . Determinants of the uptake of the full dose of diphtheria‐pertussis‐tetanus vaccines (DPT3) in Northern Nigeria: A multilevel analysis. Matern Child Health J 2009: 13: 550–558. 18607704 10.1007/s10995-008-0386-5

[26] Amin R , Li Y . NGO‐promoted women's credit program, immunization coverage, and child mortality in rural Bangladesh. Women Health 1997: 25: 71–87. 9253139 10.1300/J013v25n01_05

[27] Steele F , Diamond I , Amin S . Immunization update in rural Bangladesh: A multilevel analysis. J Roy Stat Soc 1996: 159: 289–299.

[28] Carter MR , Maluccio JA . Social capital and coping with economic shocks: International Food Policy Research Institute (IFPRI), 2002.

[29] Harpham T , De Silva MJ , Tuan T . Maternal social capital and child health in Vietnam. J Epidemiol Community Health 2006: 60: 865–871. 16973533 10.1136/jech.2005.044883 PMC2566054

[30] Nobles J , Frankenberg E . Mothers’ community participation and child health. J Health Soc Behav 2009: 50: 16–30. 19413132 10.1177/002214650905000102

[31] Cleland J , van Ginneken JK . Educational attainment and health/survival In: Heggenhougen K Quah S (eds). International Encyclopedia of Public Health. Academic Press: New York, 2008, 295–303.

[32] Bloom S , Wypij D , Das GM . Dimensions of women's autonomy and the influence on maternal health care utilization in a North Indian city. Demography 2001: 38: 67–78. 11227846 10.1353/dem.2001.0001

[33] Jejeebhoy S , Sathar Z . Women's autonomy in India and Pakistan: The influence of religion and region. Popul Dev Rev 2001: 27: 687–712.

[34] Desai S , Alva S . Maternal education and child health: Is there a strong causal relationship? Demography 1998: 35: 71–81. 9512911

[35] Dinçer MA , Kaushal N , Grossman M . Women's education: Harbinger of another spring? Evidence from a natural experiment in Turkey. World Dev 2014: 64: 243–258.

[36] Breierova L , Duflo E . The impact of education on fertility and child mortality: Do fathers really matter less than mothers? *OECD Development Centre* 2003; Working Paper No. 217.

[37] Psaki SR , Mensch BS , Chuang EM , Melnikas AJ . Evidence for Causal Links Between Education and Maternal and Child Health, Sexual and Reproductive Health, and Malaria: A Systematic Review. Population Association of America. Denver, Colorado: Population Council; 2018.

[38] Higgins J , Green SE . Cochrane Handbook for Systematic Reviews of Interventions Version 5.1.0 2011. (Available from http://handbook.cochrane.org.2018).

[39] Baird S , Ferreira FH , Özler B , Woolcock M . Relative effectiveness of conditional and unconditional cash transfers for schooling outcomes in developing countries: a systematic review. Campbell Syst Rev 2013: 9.

[40] Aloe AM , Thompson CG . The synthesis of partial effect sizes. J Soc Social Work Res 2013: 4: 390–405.

[41] Cohen J . Statistical Power Analysis for the Behavioral Sciences (2nd edn). Erlbaum Associates: Hillsdale, 1988.

[42] Lipsey MW , Wilson D . Practical Meta‐Analysis (Applied Social Research Methods). Sage Publications: Thousand Oaks, 2001.

[43] Lipsey MW , Wilson DB . Practical Meta‐Analysis. Sage Publications Inc: Thousand Oaks, 2001.

[44] Chung Y , Rabe‐Hesketh S , Choi IH . Avoiding zero between‐study variance estimates in random‐effects meta‐analysis. Stat Med 2013: 32: 4071–4089. 23670939 10.1002/sim.5821

[45] Novianti PW , Roes KC , van der Tweel I . Estimation of between‐trial variance in sequential meta‐analyses: a simulation study. Contemp Clin Trials 2014: 37: 129–138. 24321246 10.1016/j.cct.2013.11.012

[46] Viechtbauer W . Bias and Efficiency of Meta‐Analytic Variance Estimators in the Random‐Effects Model. J Educ Behav Stat 2005: 30: 261–293.

[47] Veroniki AA , Jackson D , Viechtbauer W *et al* Methods to estimate the between‐study variance and its uncertainty in meta‐analysis. Res Synth Methods 2016: 7: 55–79. 26332144 10.1002/jrsm.1164 PMC4950030

[48] Cooper H . Research Synthesis and Meta‐Analysis: A Step by Step Approach (5th edn). SAGE Publications, Inc.: Thousand Oaks, 2017.

[49] Grépin KA , Bharadwaj P . Maternal education and child mortality in Zimbabwe. J Health Econ 2015: 44: 97–117. 26569469 10.1016/j.jhealeco.2015.08.003

[50] Makate M , Makate C . The causal effect of increased primary schooling on child mortality in Malawi: Universal primary education as a natural experiment. Soc Sci Med 2016: 168: 72–83. 27639483 10.1016/j.socscimed.2016.09.003

[51] Ali FRM , Elsayed MA . The effect of parental education on child health: Quasi‐experimental evidence from a reduction in the length of primary schooling in Egypt. Health Econ 2018: 27: 649062. 10.1002/hec.3622 29237231

[52] Makate M . Education Policy and Under‐Five Survival in Uganda: Evidence from the Demographic and Health Surveys. Social Sci 2016: 5: 70.

[53] Shrestha V . Can Basic Maternal Literacy Skills Improve Infant Health Outcomes?Evidence from the Education Act in Nepal. *Towson University Department of Economics* 2016; Working Paper No. 2016‐08.

[54] Dursun B , Cesur R , Kelly IR . The value of mandating maternal education in a developing country. Natl Bur Econ Res 2017, Working Paper No. 23492.

[55] Keats A . Women's schooling, fertility, and child health outcomes: Evidence from Uganda's free primary education program. *Wesleyan University Department of Economics* 2018; Working Paper.

[56] Baird S , McIntosh C , Ozler B . When the Money Runs Out: Do cash transfers have sustained effects on human capital accumulation? 2018.

[57] De Neve J‐W , Subramanian S . Causal Effect of Parental Schooling on Early Childhood Undernutrition: Quasi‐Experimental Evidence From Zimbabwe. Am J Epidemiol 2017: 187: 82–93. 10.1093/aje/kwx195 29309520

[58] Fazlul I . The Effect of Mother's Education on Child Health: Evidence from Bangladesh. 2018.

[59] Güneş PM . The role of maternal education in child health: Evidence from a compulsory schooling law. Econ Educ Rev 2015: 47: 1–16.

[60] Maïga EW . The Impact of Mother's Education on Child Health and Nutrition in Developing Countries: Evidence from a Natural Experiment in Burkina Faso. *African Center for Economic Transformation Accra, Ghana* 2011; Working Paper.

[61] Tequame M , Tirivayi N . Higher education and fertility: Evidence from a natural experiment in Ethiopia. UNU‐Merit Working Paper Series: United Nations University; 2015 June 30.

[62] Saaka M , Galaa SZ . Relationships between wasting and stunting and their concurrent occurrence in Ghanaian preschool children. J Nutr Metab 2016: 2016: 1–11. 10.1155/2016/4654920 PMC4917721 27379184

[63] De Onis M , Blossner M , Organization WH . WHO global database on child growth and malnutrition: Geneva: World Health Organization, 1997. 10.1093/ije/dyg099 12913022

[64] Weitzman A . The effects of women's education on maternal health: Evidence from Peru. Soc Sci Med 2017: 180: 1–9. 28301806 10.1016/j.socscimed.2017.03.004 PMC5423409

[65] Wooldridge JM . Econometric Analysis of Cross Section and Panel Data. MIT press: Cambridge, 2010.

[66] Angrist JD , Krueger AB . Instrumental variables and the search for identification: From supply and demand to natural experiments. J Econ Perspect 2001: 15: 69–85.

[67] Bicego GT , Boerma JT . Maternal education and child survival: a comparative study of survey data from 17 countries. Soc Sci Med 1993: 36: 1207–1227. 8511650 10.1016/0277-9536(93)90241-u

[68] Huedo‐Medina TB , Sánchez‐Meca J , Marín‐Martínez F , Botella J . Assessing heterogeneity in meta‐analysis: Q statistic or I² index? Psychol Methods 2006: 11: 193. 16784338 10.1037/1082-989X.11.2.193

[69] Sterne JA , Egger M , Smith GD . Investigating and dealing with publication and other biases in meta‐analysis. Br Med J 2001: 323: 101–105. 11451790 10.1136/bmj.323.7304.101 PMC1120714

[70] Guillot M , Gerland P , Pelletier F , Saabneh A . Child mortality estimation: a global overview of infant and child mortality age patterns in light of new empirical data. PLoS Med 2012: 9: e1001299. 22952438 10.1371/journal.pmed.1001299 PMC3429403

[71] WHO . WHA global nutrition targets 2025: Stunting policy brief. 2014. (Available from: https://www.who.int/nutrition/topics/globaltargets_stunting_policybrief.pdf.)

[72] UNDP . Human Development Reports 2018 Statistical Update. 2018. (Available from: http://hdr.undp.org/en/2018-update) [1 Feb 2019]

