# Supplementary material for: Evidence for causal links between education and maternal and child health: systematic review
Source: Trop Med Int Health. 2019 Mar 28;24(5):504–22. doi: 10.1111/tmi.13218 (PMC6519047; doi:10.1111/tmi.13218)
Supplement: Supplementary file 1 — Table S1. List of outcomes in the 15 infant and child health papers. [file TMI-24-504-s001.docx]

**Table S1**: **List of outcomes in the 15 infant and child health papers**

| Outcome | # of papers analyzing the effect of education on each outcome | # of papers comparing OLS to more rigorous models |
| --- | --- | --- |
| 1. Neonatal death dichotomous | 3 | 1 |
| 1. Total neonates died | 2 | 2 |
| 1. **Infant mortality dichotomous*** | 6 | 3 |
| 1. Infant mortality continuous | 1 | 1 |
| 1. Total infant deaths continuous | 2 | 2 |
| 1. Total children under 5 died | 2 | 2 |
| 1. **Under 5 mortality dichotomous*** | 4 | 3 |
| 1. Under 5 mortality rate continuous | 1 | 1 |
| 1. **Child mortality dichotomous*** | 4 | 4 |
| 1. Child mortality rate continuous | 1 | 1 |
| 1. Total children died | 1 | 1 |
| 1. Height for age z score (HAZ) | 5 | 3 |
| 1. **Stunted dichotomous*** | 4 | 2 |
| 1. Total stunted children | 1 | 1 |
| 1. Weight for height z score (WHZ) | 2 | 2 |
| 1. **Wasted dichotomous*** | 4 | 2 |
| 1. Weight for age z score (WAZ) | 3 | 2 |
| Total # of outcomes | 46 | 33 |

***Forest plots and meta-analyses are included for bolded outcomes**
