# Supplementary material for: Evidence for causal links between education and maternal and child health: systematic review
Source: Trop Med Int Health. 2019 Mar 28;24(5):504–22. doi: 10.1111/tmi.13218 (PMC6519047; doi:10.1111/tmi.13218)
Supplement: Supplementary file 2 — Table S2. Risk of bias assessment results by study. [file TMI-24-504-s002.docx]

**Table S2:** Risk of bias assessment results by study

|  |  |  | **RCTs** | **Quasi-Experimental** | | | |  |  |  |  |  |
| --- | --- | --- | --- | --- | --- | --- | --- | --- | --- | --- | --- | --- |
| **Author(s)/year** | **Study Type** | **Selection Bias** | **Inclusion/**  **Exclusion Criteria** | **Regression Discontinuity** | **Natural Experiments** | **Heckman and IV** | **Panel Data** | **Sample Size** | **Confounding** | **Attrition** | **Mechanisms** | **Score** |
| Ali and Elsayed (2017) | Quasi-experimental | 1 | -- | 1 | 1 | 0 | -- | 1 | 0 | 0 | 1 | 3 |
| Baird, McIntosh and Ozler (2018) | Experimental | 1 | 1 | -- | -- | -- | -- | 1 | 1 | 1 | 1 | 6 |
| Breierova and Duflo (2003) | Quasi-experimental | 1 | -- | -- | 1 | 0 | 1 | 1 | 0 | 0 | 1 | 3 |
| De Neve and Subramanian (2017) | Quasi-experimental | 1 | -- | -- | 1 | 0 | -- | 1 | 1 | 0 | 1 | 4 |
| Dinçer, Kaushal, Grossman (2013) | Quasi-experimental | 1 | -- | -- | 1 | 1 | -- | 1 | 1 | 0 | 1 | 5 |
| Dursun, Cesur and Kelly (2017) | Quasi-experimental | 1 | -- | -- | 1 | 0 | -- | 1 | 1 | 1 | 1 | 5 |
| Fazlul (2018) | Quasi-experimental | 1 | -- | -- | 1 | 0 | -- | 1 | 0 | 0 | 0 | 2 |
| Grepin and Bharadwaj (2015) | Quasi-experimental | 1 | -- | 1 | 1 | 0 | -- | 1 | 0 | 0 | 1 | 3 |
| Gunes (2015) | Quasi-experimental | 1 | -- | -- | 1 | 0 | -- | 1 | 1 | 0 | 1 | 4 |
| Keats (2018) | Quasi-experimental | 1 | -- | 1 | 0 | 0 | -- | 1 | 0 | 0 | 1 | 3 |
| Maïga (2011) | Quasi-experimental | 1 | -- | -- | 1 | 0 | -- | 1 | 0 | 0 | 1 | 3 |
| Makate (2016) | Quasi-experimental | 1 | -- | 0 | 1 | 0 | -- | 1 | 1 | 0 | 1 | 4 |
| Makate and Makate (2016) | Quasi-experimental | 1 | -- | 1 | 1 | 0 | -- | 1 | 1 | 0 | 1 | 4 |
| Shrestha (2016) | Quasi-experimental | 1 | -- | -- | 1 | 0 | -- | 1 | 1 | 0 | 1 | 4 |
| Tequame and Tirivayi (2014) | Quasi-experimental | 1 | -- | 1 | 1 | 0 | -- | 1 | 1 | 0 | 1 | 4 |
| Weitzman (2017) | Quasi-experimental | 1 | -- | 1 | 1 | 0 | -- | 1 | 0 | 0 | 1 | 3 |

Note: -- **= not applicable**
