# Supplementary material for: Evidence for causal links between education and maternal and child health: systematic review
Source: Trop Med Int Health. 2019 Mar 28;24(5):504–22. doi: 10.1111/tmi.13218 (PMC6519047; doi:10.1111/tmi.13218)
Supplement: Supplementary file 3 — Table S3. Wasted (dichotomous). [file TMI-24-504-s003.docx]

**Table 3S Wasted (dichotomous)**

|  | **Authors (Year)** | **Country** | **Age Group** | **Education Exposure** | **Health Outcome** | **Partial correlation r: OLS models (95% CI)** | **Partial Correlation r: More rigorous models (95% CI)** | **Forest Plot** |
| --- | --- | --- | --- | --- | --- | --- | --- | --- |
|  | |  |  |  |  |  | \|  \|  \|  \| \| --- \| --- \| --- \| |  |
|  | Ali & Elsayed (2018) | Egypt | 15-49 | Grade attainment (continuous) | Wasted (dichotomous) | Not calculated | 0.001  (-0.005, 0.006) |  |
|  | De Neve & Subramanian (2017)ˠ | Zimbabwe | 37-57 | Grade attainment (continuous) | Wasted (dichotomous) | -0.05  (-0.074, -0.025) | -0.01  (-0.035, 0.015) |  |
|  | Keats (2018) | Uganda | 19-49 | Grade attainment (continuous) | Wasted (dichotomous) | Not calculated | 0.001  (-0.05, 0.052) |  |
|  | Fazlul (2018) | Bangladesh | 15-49 | Grade attainment (continuous) | Wasted (dichotomous) | -0.028  (-0.042, -0.015) | -0.011  (-0.025, 0.003) |  |
|  | Overall effect size | I²=Not calculated | Q=2.826 | tau²=0 | P-value=0.494 |  | -0.003  (-0.017, 0.011) |  |

ˠ Studies that received a risk of bias score of 4 or higher.
