# Supplementary material for: Evidence for causal links between education and maternal and child health: systematic review
Source: Trop Med Int Health. 2019 Mar 28;24(5):504–22. doi: 10.1111/tmi.13218 (PMC6519047; doi:10.1111/tmi.13218)
Supplement: Supplementary file 4 — Table S4. Weight for Height Z score continuous (WHZ). [file TMI-24-504-s004.docx]

**Table S4 Weight for Height Z score continuous (WHZ)**

|  | **Authors (Year)** | **Country** | **Age Group** | **Education Exposure** | **Health Outcome** | **Partial correlation r: OLS models (95% CI)** | **Partial Correlation r: More rigorous models (95% CI)** |
| --- | --- | --- | --- | --- | --- | --- | --- |
|  | | | |  |  |  |  |
|  | Fazlul (2018) | Bangladesh | 15-49 | Years of schooling (continuous) | WHZ (continuous) | 0.071  (0.057, 0.085) | 0.016  (0.002, 0.03) |
|  | Maiga (2011) | Burkina Faso | 19 and older | Years of schooling (log) (continuous) | WHZ (continuous) | 0.044  (-0.001, 0.088) | 0.09  (0.045, 0.135) |
|  | | |  |  |  |  |  |
