# Supplementary material for: Evidence for causal links between education and maternal and child health: systematic review
Source: Trop Med Int Health. 2019 Mar 28;24(5):504–22. doi: 10.1111/tmi.13218 (PMC6519047; doi:10.1111/tmi.13218)
Supplement: Supplementary file 5 — Table S5. Weight for Age Z score continuous (WAZ). [file TMI-24-504-s005.docx]

**Table S5 Weight for Age Z score continuous (WAZ)**

|  | **Authors (Year)** | **Country** | **Age Group** | **Education Exposure** | **Health Outcome** | **Partial correlation r: OLS models (95% CI)** | **Partial Correlation r: More rigorous models (95% CI)** |
| --- | --- | --- | --- | --- | --- | --- | --- |
|  | | | |  |  |  |  |
|  | Keats (2018) | Uganda | 19-49 | Grade attainment (continuous) | WAZ (continuous) | Not calculated | 0.019  (-0.032, 0.07) |
|  | Fazlul (2018) | Bangladesh | 15-49 | Years of schooling (continuous) | WAZ (continuous) | 0.118  (0.104, 0.132) | 0.013  (-0.001, 0.027) |
|  | Gunes (2015)ˠ | Turkey | 18-29 | Grade attainment (dichotomous 8+ years) | WAZ (continuous) | 0.064  (0.006, 0.122) | 0.048  (-0.001, 0.097) |
| ˠ Studies that received a risk of bias score of 4 or higher. | | |  |  |  |  |  |
