# Supplementary material for: Evidence for causal links between education and maternal and child health: systematic review
Source: Trop Med Int Health. 2019 Mar 28;24(5):504–22. doi: 10.1111/tmi.13218 (PMC6519047; doi:10.1111/tmi.13218)
Supplement: Supplementary file 6 — Table S6. Maternal Morbidity. [file TMI-24-504-s006.docx]

**Table S6 Maternal Morbidity**

|  | | **Authors (Year)** | **Country** | **Age Group** | **Education Exposure** | **Health Outcome** | **Partial correlation r: OLS models (95% CI)** | **Partial Correlation r: More rigorous models (95% CI)** |
| --- | --- | --- | --- | --- | --- | --- | --- | --- |
| **Preventable** | | | |  |  |  |  |  |
|  | | Weitzman (2017) | Peru | 23-30 | Grade attainment (continuous) | Maternal morbidity (fever & vaginal bleeding during preg) (dichotomous) | -0.015  (-0.044, 0.014) | -0.03  (-0.059, -0.001) |
|  | | Weitzman (2017) | Peru | 23-30 | Grade attainment (continuous) | Maternal morbidity (fever after preg) (dichotomous) | 0.015  (-0.014, 0.044) | -0.03  (-0.059, -0.001) |
| **Semi-preventable** | | | |  |  |  |  |  |
|  | | Weitzman (2017) | Peru | 23-30 | Grade attainment (continuous) | Maternal morbidity (excessive bleeding during preg) (dichotomous) | -0.022  (-0.052, 0.007) | -0.03  (-0.059, -0.001) |
|  |  | Weitzman (2017) | Peru | 23-30 | Grade attainment (continuous) | Maternal morbidity (convulsions) (dichotomous) | -0.004  (-0.034, 0.025) | -0.05  (-0.079, -0.021) |
| **Not preventable** | | | |  |  |  |  |  |
|  | | Weitzman (2017) | Peru | 23-30 | Grade attainment | Maternal morbidity (mastitis) (dichotomous) | 0.075  (0.045, 0.104) | -0.015  (-0.044, 0.014) |
