# Supplementary material for: Evidence for causal links between education and maternal and child health: systematic review
Source: Trop Med Int Health. 2019 Mar 28;24(5):504–22. doi: 10.1111/tmi.13218 (PMC6519047; doi:10.1111/tmi.13218)
Supplement: Supplementary file 7 — Table S7. Evidence in support of hypothesised mechanisms linking grade attainment and infant mortality. Table S8. Evidence in support of hypothesised mechanisms linking grade attainment and child mortality. Table S9. Evidence in support of hypothesised mechanisms linking grade attainment and child growth faltering. [file TMI-24-504-s007.docx]

**Table S7. Evidence in support of hypothesized mechanisms linking grade attainment and infant mortality**

|  | *Support for mechanism* | *No support for mechanism* |
| --- | --- | --- |
| **Sexual and Reproductive Behavior** | | |
| Age at first birth | - Dinçer, Kaushal, & Grossman^51^ (2013) - Grépin & Bharadwaj^50^ (2015) |  |
| Age at childbearing | - Keats (2018)^43^ * |  |
| Fertility/ Number of children born/Parity | - Dinçer, Kaushal, & Grossman^51^ (2013) - Grépin & Bharadwaj^50^ (2015) - Makate & Makate^49^ (2016) |  |
| Fertility preferences | - Keats (2018)^43^ |  |
| Onset of sexual activity | - Keats (2018)^43^ |  |
| Age at first marriage/ Age at first cohabitation | - Dinçer, Kaushal, & Grossman^51^ (2013) - Grépin & Bharadwaj^50^ (2015) |  |
| Age at first intercourse | - Grépin & Bharadwaj^50^ (2015) |  |
| **Health Behaviors** | | |
| Antenatal/Prenatal care | - Makate & Makate^49^ (2016) | - Ali & Elsayed^46^ (2017) - Grépin & Bharadwaj^50^ (2015) |
| Breastfeeding |  | - Makate & Makate^49^ (2016) |
| Use of modern contraception | - Keats (2018) ^43^ - Dinçer, Kaushal, & Grossman^51^ (2013) |  |
| Delivery in health facility /Medical assistant at delivery | - Keats (2018)^43^ | - Grépin & Bharadwaj^50^ (2015) |
| Child immunization | - Keats (2018)^43^ - Makate & Makate^49^ (2016) | - Grépin & Bharadwaj^50^ (2015) |
| **Maternal Health Status** |  |  |
| C-section | - Grépin & Bharadwaj^50^ (2015) | - Makate & Makate^49^ (2016) |
| Mother’s health status | - Shrestha^40^ (2016) |  |
| **Resources** | | |
| Assortative mating | - Makate & Makate^49^ (2016) | - Breierova & Duflo^48^ (2004) - Keats (2018)^43^ |
| Access to media | - Keats (2018)^43^ |  |
| Formal employment/Cash employment | - Keats (2018)^43^ |  |
| Labor force participation | - Grépin & Bharadwaj^50^ (2015) |  |
| Income |  |  |
| Wealth/Asset ownership | - Keats (2018) ^43^ - Grépin & Bharadwaj^50^ (2015) |  |
| Access to clean water | - Shrestha^40^ (2016) |  |
| Electricity | - Shrestha^40^ (2016) |  |
| Toilet | - Shrestha^40^ (2016) |  |
| Urban Migration/residence | - Keats (2018)^43^ - Grépin & Bharadwaj^50^ (2015) |  |
| Female head of household |  | - Makate & Makate^49^ (2016) |
| **Knowledge, Skills and Attitudes** | | |
| Literacy | - Keats (2018)^43^ - Makate & Makate^49^ (2016) | - Ali & Elsayed^46^ (2017) |
| Attitudes toward gender equality |  | - Dinçer, Kaushal, & Grossman^51^ (2013) |
| Bargaining Power within Household |  | - Keats^43^ (2018) |
| Opinions on domestic violence and risky behavior (smoking) |  | - Grépin & Bharadwaj^50^ (2015) |
| Knowledge of modern contraception | - Keats (2018)^43^ - Dinçer, Kaushal, & Grossman^51^ (2013) |  |

Note: Makate^43^ (2016) did not investigate mechanisms.

**Table S8. Evidence in support of hypothesized mechanisms linking grade attainment and child mortality**

|  | *Support for pathway* | *No support for pathway* |
| --- | --- | --- |
| **Sexual and Reproductive Behavior** | | |
| Age at first birth | - Dinçer, Kaushal, & Grossman^51^ (2013) - Grépin & Bharadwaj^50^ (2015) |  |
| Fertility/Parity/Number of children born | - Dinçer, Kaushal, & Grossman^51^ (2013) - Grépin & Bharadwaj^50^ (2015) - Makate & Makate^49^ (2016) |  |
| Age at first marriage/ Age at first cohabitation | - Dinçer, Kaushal, & Grossman^51^ (2013) - Grépin & Bharadwaj^50^ (2015) |  |
| Age at first intercourse | - Grépin & Bharadwaj^50^ (2015) |  |
| **Health Behaviors** | | |
| Antenatal/Prenatal care | - Makate & Makate^49^ (2016) | - Ali & Elsayed^46^ (2017) - Grépin & Bharadwaj^50^ (2015) |
| Smoking | - Dursun, Cesur & Kelly^52^ (2017) |  |
| Breastfeeding |  | - Makate & Makate^49^ (2016) |
| Use of modern contraception | - Dinçer, Kaushal, & Grossman^51^ (2013) |  |
| Delivery in health facility |  | - Grépin & Bharadwaj^50^ (2015) |
| Child immunization | - Makate & Makate^49^ (2016) | - Grépin & Bharadwaj^50^ (2015) |
| **Maternal Health Status** | | |
| C-section | - Grépin & Bharadwaj^50^ (2015) - Dursun, Cesur & Kelly^52^ (2017) | - Makate & Makate^49^ (2016) |
| Mother’s health status | - Shrestha^40^ (2016) |  |
| **Resources** | | |
| Assortative Mating | - Makate & Makate^49^ (2016) | - Breierova & Duflo^48^ (2004) |
| Labor force participation | - Grépin & Bharadwaj^50^ (2015) |  |
| Asset Ownership | - Grépin & Bharadwaj^50^ (2015) |  |
| Access to clean water | - Shrestha^40^ (2016) |  |
| Electricity | - Shrestha^40^ (2016) |  |
| Toilet | - Shrestha^40^ (2016) |  |
| Urban residence | - Grépin & Bharadwaj^50^ (2015) |  |
| Female head of household |  | - Makate & Makate^49^ (2016) |
| **Knowledge, Skills and Attitudes** | | |
| Literacy | - Makate & Makate^49^ (2016) | - Ali & Elsayed^46^ (2017) |
| Attitudes toward gender equality |  | - Dinçer, Kaushal, & Grossman^51^ (2013) |
| Women’s empowerment |  | - Grépin & Bharadwaj^50^ (2015) |
| Opinions on domestic violence and risky behavior (smoking) |  | - Grépin & Bharadwaj^50^ (2015) |
| Knowledge of modern contraception | - Dinçer, Kaushal, & Grossman^51^ (2013) |  |

Note: Makate^43^ (2016) did not investigate mechanisms.

**Table S9. Evidence in support of hypothesized mechanisms linking grade attainment and child growth faltering**

|  | *Support for mechanism* | *No support for mechanism* |
| --- | --- | --- |
| **Sexual and Reproductive Behavior** | | |
| Age at first birth | - Güneş^45^ (2015) |  |
| Age at childbearing | - Keats^43^ (2018) |  |
| Fertility/Number of children born | - Keats^43^ (2018) - Güneş^45^ (2015) |  |
| Fertility preferences | - Keats^43^ (2018) |  |
| Onset of sexual activity | - Keats^43^ (2018) |  |
| **Health Behaviors** | | |
| Prenatal care initiation | - Güneş^45^ (2015) |  |
| Smoking | - Güneş^45^ (2015) |  |
| Use of modern contraception | - Keats^43^ (2018) |  |
| Delivery in a health facility/Delivery by health professional/Medical assistant at delivery | - Keats^43^ (2018) | - Güneş^45^ (2015) |
| Child immunization | - Keats^43^ (2018) |  |
| Per capita Health Expenditures | - Maïga^44^ (2011) |  |
| **Resources** | | |
| Access to media | - Keats^43^ (2018) |  |
| Occupation |  | - Güneş^45^ (2015) |
| Labor force participation |  | - Güneş^45^ (2015) |
| Wealth/Asset ownership/Income | - De Neve & Subramanian^47^ (2017) - Keats^43^ (2018) | - De Neve & Subramanian^47^ (2017) |
| Urban Migration/Residence | - Keats^43^ (2018) - De Neve & Subramanian^47^ (2017) | - De Neve & Subramanian^47^ (2017) |
| Distance from health clinic |  | - Maïga^44^ (2011) |
| **Knowledge, Skills and Attitudes** | | |
| Literacy | - Keats^43^ (2018) | - Ali & Elsayed^46^ (2017) |
| Bargaining power within household | - Maïga^44^ (2011) | - Keats^43^ (2018) |
| Knowledge of modern contraception | - Keats^43^ (2018) |  |
| Health Knowledge |  | - Maïga^44^ (2011) |

Notes: Fazlul (2018) and Tequame & Tirivayi^54^ (2015) did not investigate mechanisms. Baird, McIntosh & Ozler (2018) investigated the direct income effect of the CCT on child growth faltering, but not explicitly as a pathway linking education to health.
