# Supplementary material for: Evidence for causal links between education and maternal and child health: systematic review
Source: Trop Med Int Health. 2019 Mar 28;24(5):504–22. doi: 10.1111/tmi.13218 (PMC6519047; doi:10.1111/tmi.13218)
Supplement: Supplementary file 8 — Data S1. Model types and formulas used for conversion to partial correlations. [file TMI-24-504-s008.docx]

***Supplement S10:*** Model types and formulas used for conversion to partial correlations

Note:

- IV here refers to independent variable, NOT instrumental variable
- DV refers to the dependent variable
- For all two-stage regressions, the second stage regressor is referenced as the IV
- $r$ refers to the partial correlation
- Equation 1.1 was applied to all results with continuous DVs to improve comparability, unless the equation didn’t properly fit the reported models. In those cases, we applied Equations 1.2.1-2.3 based on the bolded criteria below.

**Continuous DV**

- **Linear models with either Continuous or Dichotomous IVs**
  - Equation 1:
    - Equations:
      - $t=\frac{B}{{se}_{B}}$, where $t$ refers to the t-statistic
      - $r=\frac{t}{\sqrt{t^{2}+df}}$
    - Data needed:
      - T-statistic ($t$)or Unstandardized Regression Coefficient and Standard Error ($B, {se}_{B}$)
      - Residual Degrees of Freedom (sample size minus the number of predictors) ($df$)

**Dichotomous DV**

- **Linear models with dichotomous IVs**
- Equation 2: Linear models with dichotomous IV and dichotomous DV (if control group success proportion is presented)
  - Equations:
    - $a=n_{treat}(p_{control}+B)$
    - $b=n_{treat}(1-(p_{control}+B))$
    - $c=n_{control}*p_{control}$
    - $d=n_{control}(1-p_{control})$
    - $r=\frac{\left( ad \right)-(bc)}{\sqrt{(a+b)(c+d)(a+c)(b+d)}}$
  - Data needed:
    - Unstandardized Regression Coefficient ($B$)
    - Control group sample size ($n_{treat}$)
    - Treatment group sample size ($n_{control}$)
    - Control group success proportion (i.e. mean) of DV ($p_{control}$)

**Standard errors and Confidence Intervals**

- **Standard errors**
  - If only the standard error of the coefficient was available:
    - Equation 3.1:
      - ${se}_{r}=\frac{r*{se}_{B}}{B}$, where ${se}_{r}$ refers to the standard error of the Partial correlation
    - Data needed
      - Unstandardized Regression Coefficient ($B$)
      - Standard Error of the Unstandardized Regression Coefficient (${se}_{B}$)
  - If only the 95% confidence intervals for the coefficient were available:
    - Equation 3.2:
      - ${se}_{B}=\frac{{CI}_{upper}-{CI}_{lower}}{1.96}$, where ${se}_{B}$ refers to the standard error of the unstandardized regression coefficient
      - ${se}_{r}=\frac{r*{se}_{B}}{B}$, where ${se}_{r}$ refers to the standard error of the Partial correlation
    - Data needed
      - Unstandardized Regression Coefficient ($B$)
    - Confidence intervals of the Unstandardized Regression Coefficient (${CI}_{upper}$, ${CI}_{lower}$)
- **Confidence intervals**
  - The equation below can apply to regression coefficients as well as partial correlations:
    - Equation 3.3
      - $CI=B\pm{se}_{B}\cdot1.96$
